# Assessment of the Impact of Parental BMI on the Incidence of Overweight and Obesity in Children from Ukraine

**DOI:** 10.3390/jcm9041060

**Published:** 2020-04-08

**Authors:** Katarzyna Dereń, Justyna Wyszyńska, Serhiy Nyankovskyy, Olena Nyankovska, Edyta Łuszczki, Marek Sobolewski, Artur Mazur

**Affiliations:** 1 Institute of Health Sciences, Medical College of Rzeszow University, 35-959 Rzeszow, Poland; justyna.wyszynska@onet.pl (J.W.); nianksl@gmail.com (S.N.); eluszczki@ur.edu.pl (E.Ł.); 2 Centre for Innovative Research in Medical and Natural Sciences, University of Rzeszow, 35-310 Rzeszow, Poland; 3 Pediatrics Department #1, Danylo Halytsky L’viv National Medical University, 79010 Lviv, Ukraine; 4 Department of Pediatrics and Neonatology, Danylo Halytsky L’viv National Medical University, 79010 Lviv, Ukraine; lena.nyank@gmail.com; 5 Faculty of Management, Rzeszow University of Technology, 35-959 Rzeszow, Poland; mareksobol@poczta.onet.pl; 6 Institute of Medical Sciences, Medical College of Rzeszow University, 35-959 Rzeszow, Poland; drmazur@poczta.onet.pl

**Keywords:** obesity, overweight, BMI, children, parents

## Abstract

The aim of this study was to assess the impact of parental body mass index (BMI) on the risk of having an overweight or obese child in Ukraine. This study included 22,576 parents (11,288 mothers and fathers) and the same number of children (boys 48%, girls 52%) aged 6.0–18.9 years who live in Ukraine. The study was conducted in randomly selected primary, secondary and high schools of Ukraine. Body weight and height was measured in triplicate. Based on the results obtained, BMI was calculated. The analysis was carried out based on z-score values of children and BMI classification of children. Odds ratios (OR) were calculated using logistic regression analysis. For fathers, 38.1% had normal BMI, 15.9% were obese, and 45.6% were overweight. For mothers, 52.1% of those surveyed had normal BMI, 31.8% were overweight and 13.5% were classified as obese. The vast majority (72.7%) of children had normal weight, 10.2% were overweight, and 15.0% were underweight. Children of overweight fathers had a higher risk of becoming overweight (OR = 1.41). Children of obese fathers had both a greater risk of being overweight (OR = 2.04) and obese (OR = 2.56). The odds ratios indicate that children of overweight mothers had a greater risk of being overweight (OR = 1.45) and obese (OR = 1.76). Children of obese mothers had both a greater risk of becoming overweight (OR = 2.05) and obese (OR = 2.70). More often, overweight and obese parents had children who also had higher BMI.

## 1. Introduction

As in other countries, recent studies in Ukraine have indicated an increase in the incidence of obesity among children and adolescents. The threat of excessive body weight has become a problem all over the world, and obese children are more likely to remain so as adults [1,2,3,4].

The spreading epidemic of obesity is a complex problem involving a range of interactions between the environment and the genome. Factors including epigenetic changes lasting for many generations, the hypothesis of “fetal programming”, nutrition in early childhood, and parental health behaviors may affect the development of obesity during a child’s life. Environmental factors interacting in the prenatal and postnatal period overlap the genetic risk [5]. The incidence of overweight and obesity is increasing in both developed and developing countries. Most cases of overweight and obesity in children have been recorded in the developing societies of Eastern Europe and in the Middle East [6].

Ukraine is a country also affected by the problem of overweight and obesity. According to WHO (World Health Organization) data, 53.5% of adults are overweight and 21.3% are obese [7]. The situation in the population under 20 years of age is also problematic. In terms of the prevalence of overweight and obesity in adolescents 10–19 years old, 22% of boys and 12% of girls aged 11 are overweight. Among 13-year-olds, these figures are 21% for boys and 9% for girls, and among 15-year-olds they constitute 17% and 8%, respectively [8].

According to WHO, as much as 26% of children under the age of five years are overweight [9]. Genetic and environmental factors may affect this condition. Research suggests that genetic factors can affect 25–45% of obesity and this is a polygenic inheritance [10]. However, the role of these factors is very important. Studies indicate that obesity occurs in 2/3 of offspring when both parents are obese, and in 50% if one parent is obese, and only in 9% of children of normal-weight parents [11,12,13]. Obesity is twice as common in identical twins as in non-twin siblings. Additionally, the body weight of adults who grew up in foster care is consistent with the weight of their biological parents [14]. The incidence of obesity in one parent increases the risk of obesity among children in adulthood by a factor of four to five times. When obesity affects both parents, the risk increases 13-fold [15]. The development of excessive body weight is primarily influenced by non-genetic factors, i.e., mainly environmental factors which determine the development of the pathological phenotypes such as incorrect eating habits, and lack of physical activity [16,17,18,19].

The purpose of this study was to investigate the relationship between parental body weight and overweight and obesity in children and adolescents living in Ukraine.

## 2. Materials and Methods 

### 2.1. Participants 

The study was conducted in randomly selected primary, secondary, and high schools of Ukraine. Sample sizes were determined using the EPI INFO (StatCalc, Atlanta, Georgia, US) software. The research was carried out in randomly selected places in Ukraine. The study methodology has been published in detail [20].

### 2.2. Anthropometric Measurements

Height and weight were measured for each participant. These measurements were made with the subjects in their underwear and without shoes. Each measurement was taken as the mean of three consecutive measurements. The study took place at nursing clinics. In order for a test procedure to be reliable and reproducible, examinations were carried out at the same time of a day during morning hours, using the same test equipment. Assessments were performed by the same group of researchers that have extensive experience.

In children, measurements of body weight and height were performed in triplicate. Body weight was measured with the medical scale RADWAG WPT 60/150 (RADWAG, Radom, Poland) with an accuracy of ± 100 g, and height of the children with a stadiometer attached to the scales with an accuracy of ± 0.1 cm. All scales were tared using a standardized 1 kg weight. Mean values of height and weight were obtained from three measurements in order to calculate body mass index (BMI) according to the formula: BMI = body weight in kg/height in m^2^. BMI z-score was also calculated. International Obesity Task Force (IOTF) criteria were used in our study to define childhood underweight, normal weight, overweight and obesity [21]. Parents were asked to provide their body weight and body height. Parental BMI was calculated as underweight (BMI <18.5), normal (18.5 < BMI < 24.9), overweight (BMI ≥ 25–29.9) and obese (BMI ≥ 30).

### 2.3. Statistical Analysis

In order to determine whether children of both obese (or overweight) parents are at higher risk of developing BMI disorders than in the case when obesity (or overweight) occurs only in one parent, an analysis was carried out based on calculating z-score values of children (two-factor analysis of variance was used) with BMI classification. Odds ratios (OR) were calculated using logistic regression analysis. In addition, other methods of statistical analysis were also used such as Spearman’s rank correlation coefficient or the Kruskal-Wallis test. Statistical analyses were performed using the STATISTICA 10.0 (StatSoft, Inc., Tulsa, OK, USA) and EXCEL 2010 software (Microsoft, Redmond, USA). Statistically significant correlations and differences are denoted by * or *** (for *p* ≤ 0.05; and *p* ≤ 0.001 respectively).

### 2.4. Ethics

Written informed consent was obtained from parents and children before participation in the study. The study was approved by the Bioethics Committee of the Medical Department of the University of Rzeszów, decision no 2015/12/15 on 2 December 2015, and it was conducted in accordance with ethical standards stated in the Declaration of Helsinki.

## 3. Results

Children (15,456) from primary and secondary schools, together with their parents, qualified for this study. Based on the consent obtained from parents, the study was conducted in 13,739 children aged 6.0–18.9 years. For 11,288 of these children, parental BMI data was also available. The mean age of the children was 11.5 years. The mean age of girls (11.43 years) and boys (11.56 years) did not differ significantly. The differences in the number of examined groups of girls and boys were not statistically significant.

Descriptive BMI classification allowed us to determine that only 38% of the fathers in the study group had a normal BMI (Table 1). Every sixth male parent was obese, and nearly half were overweight. Every second mother surveyed had a normal BMI, every third was overweight and every seventh was classified as obese. The vast majority (almost ¾) of children had normal weight. Every tenth child was overweight and every sixth underweight.

**Figure 1:**
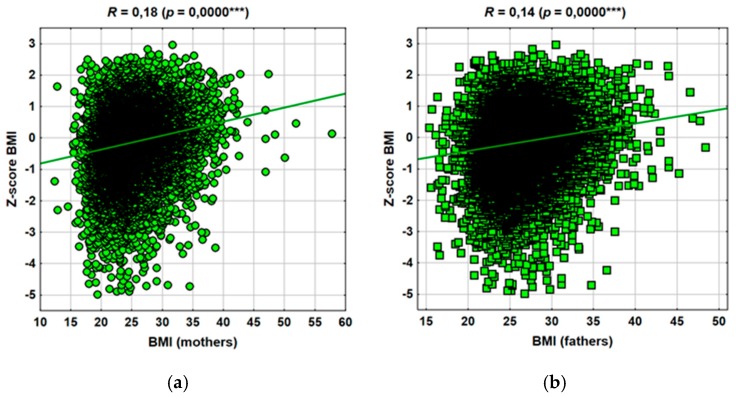
Spearman’s rank correlation between (**a**) mothers’ body mass index (BMI) and (**b**) fathers’ BMI with z-score BMI of children.

Figure 1 shows the results of the analysis of the relationship between parental BMI and the children’s BMI (z-score) classification. Both the fathers’ and mothers’ BMI (in this case more) had a statistically significant effect on the child’s BMI. This correlation was not strong, but the relationship was obvious.

The BMI of parents had a statistically significant influence on the BMI of children (Table 2). More often, overweight and obese parents had children who also had a higher BMI. Underweight parents had lower mean z-scores than parents who had a normal body weight. Children of overweight parents had higher z-scores than parents with normal BMI, and children of overweight parents had the highest z-scores.

Among overweight fathers, every tenth child was also overweight (Table 3). In the case of obesity, this relationship was even higher (over 14% of children were overweight and 4% obese). In the group of men with normal weight, the percentage of overweight children was approximately 8%. This relationship was highly statistically significant. In the case of obese mothers, as many as 15% of children were overweight (this indicator in the group of women with normal BMI was 8%). Underweight mothers and fathers also had a frequent occurrence of underweight children.

The analysis is supplemented with odds ratio values for overweight, obesity and overweight or obesity in children depending on the BMI of the father and mother. To make the results of the analysis more reliable, a small group of underweight fathers and mothers were included in the group with normal BMI. The odds ratios indicate that children of overweight fathers have a higher risk of being overweight (OR = 1.41; lower limit of 95% C.I.- 1.22) (Table 4). Children of obese fathers did not have a greater risk of obesity compared to children of overweight fathers. Children of obese fathers had not only a greater risk of becoming overweight (OR = 2.04; lower limit 95% C.I.- 1.71), but also becoming obese (OR = 2.56; lower limit 95% C.I.- 1.85). Odds ratios indicated that children of overweight mothers had a higher risk of being overweight (OR = 1.45; lower limit of 95% C.I.- 1.26), as well as obese (OR = 1.76; lower limit of 95% C.I.- 1 31). Children of obese mothers had not only a higher risk of becoming overweight (OR = 2.05; lower limit 95% C.I.- 1.74), but also obese (OR = 2.70; lower limit 95% C.I.- 1.93) (Table 4).

So far, the influence of the mothers’ and fathers’ BMI on weight disorders in children have been reported separately. It is also interesting to determine whether the incidence of high BMI in mothers and fathers at the same time “cumulates”, i.e., whether the children of both parents with obesity (or overweight) are not more exposed to the occurrence of a BMI disorder. Table 5 summarizes the mean BMI level of children against the BMI classification of mothers and fathers. The analysis of mean values shows that the incidence of overweight (obesity) in both parents increased the risk of such disorders in children.

**Figure 2:**
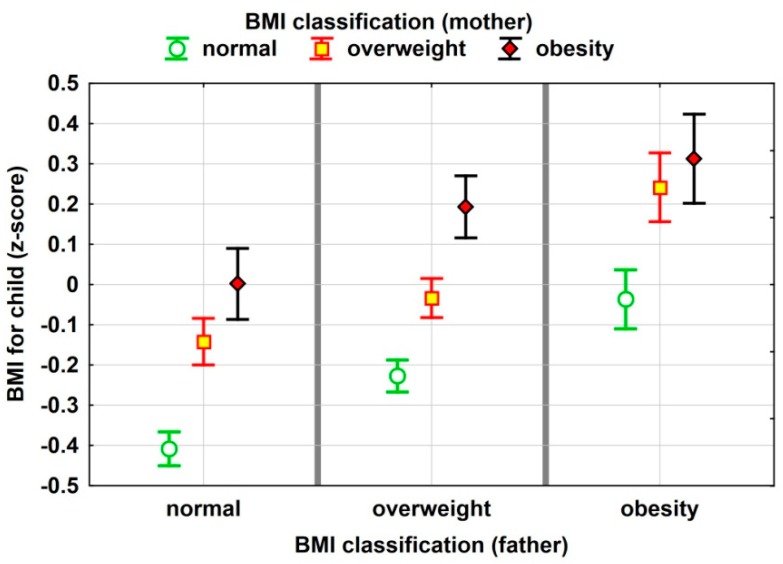
Mean values with 95% confidence intervals for BMI z-scores in relation to the BMI classification of both parents.

Figure 2 aids in depicting this relationship by presenting mean values together with 95% confidence intervals for BMI z-scores in relation to the BMI classification of both parents.

The results of the two-factor analysis of variance used to test the significance of the influence of selected factors on children’s z-score (and interaction between them) are presented below. The impact of fathers’ and mothers’ BMI classification on the children’s z-score is statistically significant, while the lack of interaction between these factors means that the effects of excessive BMI in father and mother are cumulative. Here are assessments of the statistical significance of individual effects: BMI classification (father) (*p* = 0.0000 ***); BMI classification (mother) (*p* = 0.0000 ***); interaction of fathers’ and mothers’ BMI (*p* = 0.3049).

Table 6 presents the prevalence of overweight or obesity in children depending on the BMI classification of both parents. A thorough analysis of the table below leads to the conclusion that the incidence of overweight or obesity among children is affected by the BMI of both parents, and that the strength of these interactions is cumulative.

The significance of the influence of the father and mother’s BMI classification on the incidence of overweight and obesity and the lack of interaction between them, performed using a logistic regression model, was assessed (Table 7).

The lack of significance of the impact of the interaction between the mother and father’s BMI classification on children’s BMI means that the respective odds ratios obtained for the mother and father’s BMI separately can be multiplied by assessing their combined impact on the children’s BMI.

The assessment of the odds ratio of the incidence of body mass disorders in children relative to mother and father’s BMI classification based on two-factor logistic regression models is presented in Table 8. These results were similar to those obtained during the one-way analysis. Except for the risk of obesity in children of overweight fathers, all effects were statistically significant (this is the case when the lower limit of the 95% confidence interval exceeds 1). The influence of the father’s or mother’s obesity on the incidence of overweight or obesity in a child was the strongest (odds ratios are 2.03 and 2.07, respectively). It should be emphasized once again that in order to estimate the simultaneous impact of the incidence of overweight or obesity in both parents, the appropriate odds ratios should be multiplied by one another because the model does not have an interaction factor, so the effects for each parent “add up.”

## 4. Discussion

The present study is the first attempt to examine the relationship between the BMI status in a large population of Ukrainian children and adolescents and parental BMI. It was observed that children from families of obese parents were significantly more likely to be obese than children of parents with normal body weight. Both genetic and environmental factors contribute to obesity in children. Some environmental factors, including overweight and obesity in parents, a shared lifestyle, shared eating habits, and socioeconomic status may be associated with overweight and obesity in children [22]. Families with low socioeconomic status have less access to healthy food, which is why consumption of high-calorie foods in such families is higher [23]. Studies show that a mother’s working time also affects the incidence of overweight in children. Children of full-time working mothers are more likely to be overweight [24].

Parental body weight is an important variable in overweight and obese children and adolescents [25]. In our study, obese parents were more likely to have overweight or obese children than parents with normal body weight. Studies confirm that parental obesity is a risk factor in the development of childhood obesity [26]. Kumar et al. [27] demonstrated that 32.8% of obese children and 1.9% of normal-weight children had obese parents. The influence of obesity of one or both parents on the development of child obesity was also found by Nordyńska-Sobczak et al. [28] and Semmler et al. [29]. However, they only examined children during puberty, i.e., when the risk of obesity is the greatest. Children aged one to five years were examined by Whitaker et al. [30]. He demonstrated a risk of overweight in children when their parents are obese. Czerwonogradzka-Senczyna et al. [31] demonstrated that the occurrence of obesity in at least one parent had a significant impact on the occurrence of excessive body weight in offspring. An interesting relationship between childhood obesity in parents and their children’s body mass index was observed by Li et al. [32]. Increased weight gain in parents in childhood or adolescence was associated with the risk of obesity and high BMI in their children. Carnell et al. [33] determined the risk of overweight among twins raised in families where one or two parents were obese. These families were then compared to families with a normal-weight father and mother. During seven years, they observed a doubling of overweight in children of parents with obesity, and a decrease in its occurrence in the control group. Davison et al. [34] showed that among families with obesity, the level of consumption of high-calorie products is much higher than in families with normal body weight. They also found less physical activity among parents with obesity and their children than in the control group. Research also indicates that overweight in children is strongly associated with obesity not only among their parents but also among their grandparents [35].

Overweight parents are considered a risk factor for overweight or obesity in children. Genes and the shared environment in which they live are of great importance in terms of this relation [34]. Children usually imitate their parents. Therefore, eating habits and family lifestyle can have an impact on children’s eating habits including abnormal eating habits of parents, low physical activity and extended TV screen time [22]. A study involving children and parents from Iran confirms that parental obesity is a factor in childhood and adolescent obesity. Therefore, it is important to prevent obesity in children who have one or more obese parents [36].

Our study is the first nationwide study conducted on a very large sample to establish the relationship between the incidence of overweight and obesity in children and the body weight of parents. However, the study has limitations. Since our study has a cross-sectional character, we cannot conclude a cause-and-effect relationship. Without longitudinal data, it is not possible to establish a true cause and effect relationship, so the relationship between parental body weight and child’s BMI should be confirmed in prospective studies. Limitations also include reporting height and weight by parents which may have led to a reduction in the accuracy of the data. However, many studies have demonstrated that self-reporting of body weight and body height is reliable [37,38]. Another limitation of this study is that data about demographics, prenatal course, parental chronic diseases was not collected. In the future, the study should be expanded to include more data on ethnicity, socioeconomic status, and family size.

## 5. Conclusions

In conclusion, this study emphasizes the importance of parental body weight as a factor contributing to an increase in overweight and obesity among children and adolescents, as well as the need to implement family prevention programs.

## Figures and Tables

**Table 1:** BMI classification of parents and children.

	Father		Mother		Child	
BMI Classification	*n*	%	*n*	%	*n*	%
Underweight	45	0.40	294	2.60	1697	15.00
Normal Weight	4305	38.10	5880	52.10	8208	72.70
Overweight	5146	45.60	3588	31.80	1146	10.20
Obese	1792	15.90	1526	13.50	237	2.10

**Table 2:** Z-score classification depending on the body mass index (BMI) of fathers and mothers.

BMI Classification of Parents	Fathers					Mothers				
	BMI z-Score of Children									
	Mean	Me	Std. dev.	*c*_25_	*c*_75_	Mean	Me	Std. dev.	*c*_25_	*c*_75_
Underweight	−0.76	−0.63	1.23	−1.60	0.29	−0.60	−0.58	1.35	−1.41	0.27
Normal	−0.28	−0.19	1.09	−0.94	0.43	−0.26	−0.19	1.08	−0.89	0.46
Overweight	−0.11	−0.04	1.06	−0.72	0.61	−0.02	0.06	1.05	−0.64	0.67
Obesity	0.13	0.21	1.08	−0.53	0.87	0.16	0.20	1.03	−0.44	0.87
*p*	0.0000 ***					0.0000 ***				

*p*—test probability values were calculated using the Kruskal-Wallis test; *p* < 0.001 (***); 25th and 75th percentile, first and third quartiles.

**Table 3:** BMI classification of children depending on classification of parent’s BMI.

BMI Classification of Children	BMI Classification (Father) (*p* = 0.0000 ***)				BMI Classification (Mother) (*p* = 0.0000 ***)			
	Underweight *n* (%)	Normal *n* (%)	Overweight *n* (%)	Obesity *n* (%)	Underweight *n* (%)	Normal *n* (%)	Overweight *n* (%)	Obesity *n* (%)
Underweight	16 (35.6)	795 (18.5)	706 (13.7)	180 (10.0)	93 (31.6)	1011 (17.2)	449 (12.5)	144 (9.4)
Normal	28 (62.2)	3100 (72.0)	3806 (74.0)	1274 (71.1)	171 (58.2)	4307 (73.2)	2641 (73.6)	1089 (71.4)
Overweight	1 (2.2)	337 (7.8)	545 (10.6)	263 (14.7)	23 (7.8)	480 (8.2)	408 (11.4)	235 (15.4)
Obesity	0 (0.0)	73 (1.7)	89 (1.7)	75 (4.2)	7 (2.4)	82 (1.4)	90 (2.5)	58 (3.8)

*p*—test probability value calculated using the chi-square independence test; *p* < 0.001 (***).

**Table 4:** Odds ratio values for overweight, obesity and overweight or obesity in children depending on the BMI classification of the father and mother (overweight vs. normal and obesity vs. normal).

BMI Classification	BMI Classification (Father)					BMI Classification (Mother)				
	Normal	Overweight		Obesity		Normal	Overweight		Obesity	
	*n* (%)	*n* (%)	OR	*n* (%)	OR	*n* (%)	*n* (%)	OR	*n* (%)	OR
Overweight	338 (7.8)	545 (10.6)	1.41 (1.22–1.62) *	263 (14.7)	2.04 (1.72–2.42) *	503 (8.1)	408 (11.4)	1.45 (1.26–1.66) *	235 (15.4)	2.05 (1.74–2.42) *
Obesity	73 (1.7)	89 (1.7)	1.03 (0.75–1.41)	75 (4.2)	2.56 (1.85–3.55) *	89 (1.4)	90 (2.5)	1.76 (1.31–2.36) *	58 (3.8)	2.70 (1.93–3.78) *
Overweight/Obesity	411 (9.4)	634 (12.3)	1.35 (1.18–1.54) *	338 (18.9)	2.23 (1.91–2.60) *	592 (9.6)	498 (13.9)	1.52 (1.34–1.73) *	293 (19.2)	2.24 1.92–2.61) *

OR—Odds ratios; *p* < 0.05 (*).

**Table 5:** Mean BMI z-score of children in relation to the BMI classification of mothers and fathers.

BMI Classification (Father)	BMI Z-Score											
	BMI Classification (Mother)											
	Normal				Overweight				Obesity			
	*n*	$\bar{x}$	Me	*s*	*n*	$\bar{x}$	Me	*s*	*n*	$\bar{x}$	Me	*s*
Normal	2662	−0.41	−0.32	1.12	1195	−0.14	−0.04	1.03	493	0.00	0.03	1.00
Overweight	2719	−0.23	−0.16	1.06	1765	−0.03	0.04	1.04	662	0.19	0.22	1.01
Obesity	793	−0.04	0.01	1.05	628	0.24	0.30	1.09	371	0.31	0.36	1.09

$\bar{x}$—Mean; Me—Median; s—standard deviation.

**Table 6:** Prevalence of overweight and obesity in children depending on the BMI classification of both parents.

BMI Categories	BMI Classification (Mother)								
	Normal			Overweight			Obesity		
	BMI Classification (Father)								
	Normal	Overweight	Obesity	Normal	Overweight	Obesity	Normal	Overweight	Obesity
Overweight	7%	9%	11%	9%	11%	17%	11%	16%	20%
Obesity	1%	1%	3%	2%	2%	5%	3%	3%	6%
Overweight/Obesity	8%	10%	13%	10%	13%	22%	14%	19%	25%

**Table 7:** Assessment of the statistical significance of individual effects in logistic regression models.

Dependent Variable	Overweight	Obesity	Overweight or Obesity
**BMI Classification (Father)**	*p* = 0.0000 ***	*p* = 0.0000 ***	*p* = 0.0000 ***
**BMI Classification (Mother)**	*p* = 0.0000 ***	*p* = 0.0000 ***	*p* = 0.0000 ***
**BMI Classification (Interaction)**	*p* = 0.6994	*p* = 0.5227	*p* = 0.2984

*p* < 0.001 (***).

**Table 8:** Assessment of the odds ratio of the incidence of body mass disorders in children relative to mother and father’s BMI classification based on three two-factor logistic regression models (separately determined for incidence of overweight, obesity and overweight or obesity).

Independent Factors	Effect	Prevalence Among Children		
		Overweight	Obesity	Overweight or Obesity
		OR (95% C.I.)		
BMI Classification (Father)	obesity vs. normal	1.87 (1.58–2.23) *	2.26 (1.62–3.15) *	2.03 (1.73–2.37) *
	overweight vs. normal	1.36 (1.18–1.57) *	0.98 (0.72–1.34)	1.30 (1.14–1.48) *
BMI Classification (Mother)	obesity vs. normal	1.91 (1.62–2.26) *	2.41 (1.72–3.39) *	2.07 (1.77–2.42) *
	overweight vs. normal	1.38 (1.21–1.59) *	1.68 (1.25–2.26) *	1.45 (1.28–1.65) *

OR—odds ratio; C.I.—confidence interval. *p* < 0.05 (*).

## Data Availability

The data analyzed during this study are available at the correspondence author e-mail address: kderen@ur.edu.pl.

## References

[1] (2017). References Obesity Update 2017. http://www.oecd.org/health/obesity-update.htm.

[2] WHO (2014). Global Status Report on Noncommunicable Diseases 2014.

[3] Gwozdz W., Reisch L.A., Thøgersen J. (2015). Obesity, sustainability and public health. Handbook of Research on Sustainable Consumption.

[4] Procter K.L. (2007). The Aetiology of childhood obesity: A review. Nutr. Res. Rev..

[5] Burke V., Beilin L.J., Dunbar D. (2001). Family lifestyle and parental body mass index as predictors BMI in Australian children: A longitudinal study. Int. J. Obes..

[6] Kelishadi R. (2007). Childhood overweight, obesity, and the metabolic syndrome in developing countries. Epidemiol. Rev..

[7] Report on Modelling Adulthood Obesity Across the WHO European Region, Prepared by Consultants (led by Marsh, T. and Colleagues) for the WHO Regional Office for Europe in 2013. http://www.euro.who.int/__data/assets/pdf_file/0019/243334/Ukraine-WHO-Country-Profile.pdf?ua=1.

[8] (2012). Social Determinants of Health and Well-Being Among Young People: Health Behaviour in School-aged Children (HBSC) Study: International Report from the 2009/2010 Survey. http://www.euro.who.int/__data/assets/pdf_file/0003/163857/Social-determinants-of-health-and-well-being-among-young-people.pdf.

[9] (2015). Nutrition Landscape Information System (NLis) Country Profile: Ukraine. http://apps.who.int/nutrition/landscape/report.aspx?iso=UKR&rid=1620&goButton=Go.

[10] Kottke T.E., Wu L.A., Hoffman R.S. (2003). Economic and psychological implications of the obesity epidemic. Mayo Clin. Proc..

[11] Bryl W., Hoffman K., Miczke A., Pupek-Musialik D. (2006). Otyłość w młodym wieku—Epidemiologia, konsekwencje zdrowotne, konieczność prewencji. Obesity at a young age—Epidemiology, health consequences, the need for prevention. Doctor’s guide. Przew. Lek..

[12] Obuchowicz A. (2005). Epidemiologia nadwagi i otyłości—Narastającego problemu zdrowotnego w populacji dzieci i młodzieży. Epidemiology of overweight and obesity—A growing health problem in the population of children and adolescents. Endokrynol. Otyłość Zaburz. Przem. Materii.

[13] Szatkowska A., Bodalski J. (2003). Otyłość u dzieci i młodzieży. Obesity in children and adolescents. Przew. Lek..

[14] Stunkard A.J., Sørensen T.I., Hanis C., Teasdale T.W., Chakraborty R., Schull W.J., Schulsinger F. (1986). An adoption study of human obesity. N. Engl. J. Med..

[15] Pietrzykowska E., Wierusz-Wysocka B. (2008). Psychologiczne aspekty nadwagi, otyłości i odchudzania się. Psychological aspects of overweight, obesity and weight loss. Pol. Merk. Lek..

[16] Jodkowska M., Oblacińska A. (2007). Otyłość u polskich nastolatków. Epidemiologia, styl życia, samopoczucie. Raport z badań uczniów gimnazjów w Polsce. Obesity in Polish teenagers. Epidemiology, lifestyle, well-being. Report on Studies of Junior High School Students in Poland.

[17] Beyerlein A., Toschke A.M., Schaffrath Rosario A., von Kries R. (2011). Risk factors for obesity: Further evidence for stronger effects on overweight children and adolescents compared to normalweight subjects. PLoS ONE.

[18] Starzyk J., Wojcik M., Nazim J. (2009). Czy istnieje zespoł metaboliczny u dzieci i młodzieży? Is there a metabolic syndrome in children and adolescents?. Przegl. Lek..

[19] Lin K.W., Lam C. (2011). Screening for obesity in children and adolescents. Am. Fam. Phys..

[20] Deren K., Nyankovskyy S., Nyankovska O., Łuszczki E., Wyszyńska J., Sobolewski M., Mazur A. (2018). The prevalence of underweight, overweight and obesity in children and adolescents from Ukraine. Sci. Rep..

[21] Cole T.J., Bellizzi M.C., Flegal K.M., Dietz W.H. (2000). Establishing a standard definition for child overweight and obesity worldwide: International survey. Brit. Med. J..

[22] Jiang M.H., Yang Y., Guo X.F., Sun Y.X. (2013). Association between child and adolescent obesity and parental weight status: A cross-sectional study from rural North China. J. Int. Med. Res..

[23] Irala-Estevez J.D., Groth M., Johansson L., Oltersdorf U., Prattala R., Martinez-Gonzalez M.A. (2000). A systematic review of socio-economic differences in food habits in Europe: Consumption of fruit and vegetables. Eur. J. Clin. Nutr..

[24] Meyer S.C. (2016). Maternal employment and childhood overweight in Germany. Econ. Hum. Biol..

[25] Shafaghi K., Shariff Z.M., Taib M.N., Rahman H.A., Mobarhan M.G., Jabbari H. (2014). Indeks masy ciała rodzica jest związany z nadwagą nastolatków i otyłością w Mashhadzie w Iranie. Parent’s body mass index is associated with overweight adolescents and obesity in Mashhad, Iran. Asia Pac. J. Clin. Nutr..

[26] Agras W.S., Hammer L.D., McNicholas F., Kraemer H.C. (2004). Risk factors for childhood overweight: A prospective study from birth to 9.5 years. J. Pediatr..

[27] Kumar S., Raju M., Gowda N. (2010). Influence of parental obesity on school children. Indian J. Pediatr..

[28] Nordyńska-Sobczak M., Małecka-Tendera E., Klimek K. (1999). Czynniki ryzyka otyłości u dzieci w wieku pokwitaniowym. Risk factors for obesity in children at pubertal age. Pediatr. Pol..

[29] Semmler C., Ashcroft J., van Jaarsveld C.H., Carnell S., Wardle J. (2009). Development of overweight in children in relation to parental weight and socioeconomic status. Obesity.

[30] Whitaker R.C., Wright J.A., Pepe M.S., Seidel K.D., Dietz W.H. (1997). Predicting obesity in young adulthood from childhood and parental obesity. N. Engl. J. Med..

[31] Czerwonogrodzka-Senczyna A., Kryńska P., Majcher A., Rumińska M., Jeznach-Steinhagen A., Pyrżak B. (2014). Wpływ czynników środowiskowych na występowanie otyłości u dzieci do 7 roku życia. Impact of environmental factors on the incidence of obesity in children up to 7 years of age. Endokrynol. Pediatr..

[32] Li C., Goran M.I., Kaur H., Nollen N., Ahluwalia J.S. (2007). Developmental trajectories of overweight during childhood: Role of early life factors. Obesity.

[33] Carnell S., Haworth C.M., Plomin R., Wardle J. (2008). Genetic influence on appetite in children. Int. J. Obes. (Lond).

[34] Davison K.K., Birch L.L. (2002). Obesogenic families: Parents’ physical activity and dietary intake patterns predict girls’ risk of overweight. Int. J. Obes..

[35] Davis M.M., McGonagle K., Schoeni R.F., Stafford F. (2008). Grandparental and Parental Obesity Influences on Childhood Overweight: Implications for Primary. J Am. Board Fam. Med..

[36] Bahreynian M., Qorbani M., Khaniabadi B.M., Motlagh M.E., Safari O., Asayesh H., Kelishadi R. (2017). Association between Obesity and Parental Weight Status in Children and Adolescents. J. Clin. Res. Pediatr. Endocrinol..

[37] Lipsky L.M., Haynie D.L., Hill C., Nansel T.R., Li K., Liu D., Iannotti R.J., Simons-Morton B. (2019). Accuracy of self-reported height, weight, and BMI over time in emerging adult. Am. J. Prev. Med..

[38] Kee C.C., Lim K.H., Sumarni M.G., Teh C.H., Chan Y.Y., Nuur Hafizah M.I., Cheah Y.K., Tee E.O., Ahmad Faudzi Y., Amal Nasir M. (2017). Validity of self-reported weight and height: A cross-sectional study among Malaysian adolescents. BMC Med. Res. Methodol..

